# Monitoring Salivary Levels of Interleukin 1 Beta (IL-1*β*) and Vascular Endothelial Growth Factor (VEGF) for Two Years of Orthodontic Treatment: A Prospective Pilot Study

**DOI:** 10.1155/2021/9967311

**Published:** 2021-05-24

**Authors:** Hülya Çevik-Aras, Fadimana Isik-Altun, Hatice Kilic-Tok, Julia Naoumova

**Affiliations:** ^1^Department of Oral Medicine and Pathology, Institute of Odontology, University of Gothenburg, Sweden; ^2^Public Dental Service, Gothenburg, Region Västra Götaland, Sweden; ^3^Specialist Clinic of Orthodontics, Gothenburg, Public Dental Service, Region Västra Götaland, Sweden

## Abstract

**Results:**

A gradual increase in IL-1*β* and VEGF was observed at alignment, reaching significance at space closure (*p* = 0.002 and *p* = 0.025, respectively). At finishing, both IL-1*β* and VEGF declined, however, without reverting to baseline values (*p* = 0.172 and *p* = 0.207, respectively). Bland-Altman analysis showed the agreement between IL-1*β* and VEGF in terms of a systematic increase, with a higher percentage difference for VEGF.

**Conclusions:**

The salivary levels of both IL-1*β* and VEGF increased following orthodontic treatment and reached their peaks during the treatment stage of space closure. This novel approach provides a hint on how and when to sample saliva during orthodontic treatment to analyse bone remodelling.

## 1. Introduction

The saliva is a biological fluid that has numerous functions in the oral cavity [1]. Recent developments have increased the use of the saliva as a diagnostic fluid for monitoring the overall systemic health of an individual as the content of the saliva changes continuously based on the physiological status of the body [2]. Various cytokines and growth factors found in the saliva have been suggested to monitor inflammation and the course of disease [3, 4]. Vascular endothelial growth factor (VEGF) and interleukin 1 beta (IL-1*β*) are found in the saliva. VEGF plays an important role in angiogenesis, bone formation, wound healing, and renewal of the oral epithelium [5–7]. IL-1*β* is a proinflammatory cytokine, and elevated levels of IL-1*β* can occur in many different processes in the body as an inflammatory response, including bone remodelling and wound healing [8, 9].

Orthodontic force applied during fixed appliance treatment causes an inflammatory process in the periodontal ligament (PDL) that initiates chain reactions that ultimately lead to bone remodelling, resulting in tooth movement [10]. This force initiates different signal cascades through different cellular responses, thus initiating the remodelling process [11, 12]. In these cellular responses, IL-1*β* and VEGF are strongly associated with inflammatory processes and their levels affect the biological processes during the orthodontic treatment [12, 13]. In addition, VEGF has the ability to participate in the regulation of bone metabolism and wound healing in orthodontic treatment [14]. IL-1*β* belongs to the proinflammatory group of cytokines with the role to stimulate bone resorption [15]. Several studies have shown a strong correlation between IL-1*β* activity and bone remodelling [11, 12, 15, 16].

Gingival crevicular fluid, present in the gingival sulcus, is continuously secreted by the underlying connective tissue into the oral cavity [17]. Previous studies have shown increased levels of IL-1*β* in the gingival crevicular fluid (GCF) during orthodontic treatment [18]. However, GCF has not been widely used in clinical research due to a number of limitations, namely, the sensitive method requiring good calibration by the clinician collecting the GCF, the amount of collected fluid absorbed by the filter paper being too small and difficult to analyse, the excessive time needed, and the difficulty of using it as a chair-side method in the clinics [9].

Orthodontic tooth movement is a result of continuous inflammation followed by bone resorption and new bone formation. Following those active changes would give a better understanding of the biological processes during ortodontic treatment [10]. However, there are no studies monitoring the levels of cytokines and growth factors during different stages of orthodontic treatment. Therefore, this prospective pilot study was designed as a novel approach to monitor the continuous changes in VEGF and IL-1*β* levels in the saliva during the different stages of orthodontic treatment: baseline, alignment, space closure, and finishing.

Saliva analysis is a simple and fast method that can be used to examine the ongoing inflammatory processes associated with bone turnover during orthodontic tooth movement. The current study is aimed at showing how VEGF and IL-1*β* levels are associated with the different stages of orthodontic treatment.

## 2. Materials and Methods

### 2.1. Study Setting and Eligibility Criteria

This prospective pilot study consists of consecutively recruited individuals (*N* = 9) who were treated at the Specialist Orthodontic Clinic in Gothenburg, Sweden. All the participants were females between 15 and 20 years of age and had Angle Class II:1 (overjet ≥ 5 mm) malocclusion with moderate to severe crowding (≥6 mm) that required two maxillary and two mandibular premolar extractions and fixed appliance treatment. Only healthy patients without any medication were included. Low caries activity was registered by the general dentist using radiological and clinical examinations prior to the orthodontic treatment. Good oral hygiene was achieved before and also during the orthodontic treatment.

All patients received written and oral information about the study and written consent was signed before the start of the study. The Swedish Ethical Review Authority approved the study protocol (reg.no. 2020-03047).

### 2.2. Orthodontic Treatment

The orthodontic treatment consisted of four stages: (1) baseline: diagnosis, treatment planning, and tooth extractions; (2) alignment: start with fixed appliance treatment; (3) space closure: closure of extraction spaces and sagittal correction; and (4) finishing. The duration of the four stages was recorded for each patient.

All patients had conventional fixed appliances (MBT system) on all erupted permanent teeth in the maxillary and the mandible for approximately two years. The orthodontic wires (SMART, Beijing Smart Technology Co., Ltd.) used for alignment and finishing were 0.016^″^ HANT (heat-activated nickel-titanium, NiTi thermal wires, SMART hant) and 0.019^″^ × 0.025^″^ HANT (NiTi thermal wires, Smart hant). For space closure and sagittal correction, 0.019^″^ × 0.025^″^ SS (stainless steel, Smart SS) with e-links and CL II elastics (3.5-4.5 oz, size 3/16^″^) were used. Extraction spaces were measured *in vivo* to the nearest 0.5 mm at every appointment; i.e., every 6-8 weeks, by the same orthodontist using a caliper (Dentaurum, Ispringen, Germany).

### 2.3. Saliva Sampling and Handling

A total of 134 stimulated and 134 unstimulated saliva samples were collected from nine female patients undergoing orthodontic treatment by the same orthodontist for about two years. Saliva samples were collected before tooth extractions, i.e., at baseline and at recalls with 6-8 weeks in between for about two years. All subjects were told to refrain from eating, drinking, or carrying out any oral hygiene procedures for at least one hour before the collection of saliva. For unstimulated saliva, the subjects were asked to expectorate into a 50 ml tube until 5 ml of saliva had been collected. A timer was used to record the sampling time of approximately five minutes. For stimulated saliva, the patients were instructed to chew paraffin wax while continuously gathering all the new saliva into a 50 ml tube until 5 ml of saliva had been collected. The sampling time of approximately three minutes was recorded. After that, the volume of saliva was measured for each individual and calculated as the secretory rate (ml/min). Protease inhibitor cocktail tablets (Sigma-Aldrich, S8830; one tablet diluted in 4 ml distilled water and used 25 *μ*l/ml) and EDTA (Sigma-Aldrich, 2 mM) were immediately added to minimise protein degradation. The samples were immediately stored at −80°C pending analysis. The samples did not undergo centrifugation, as this would have led to loss of proteins [19].

### 2.4. Sandwich ELISA

An enzyme-linked immunosorbent assay was used to determine the concentration of IL-1*β* in stimulated saliva and VEGF in unstimulated saliva according to the manufacturer's instructions (R&D systems, USA), except in the case of pretreatment of unstimulated saliva samples with the anionic detergent sodium dodecyl sulphate (SDS, Sigma-Aldrich, 0.02%) in the detection of VEGF. SDS was used to dissociate the mucin particles, as described in our previous study for quantitative detection of salivary cytokines [19]. Unstimulated saliva samples were then incubated for 20 min in SDS (50 *μ*l for 200 *μ*l saliva) and placed on a 96-microwell plate in a duplicate dilution series (from 1/2 to 1/4). The detection of IL-1*β* was made in stimulated saliva samples that were incubated on a 96-microwell plate in a duplicate dilution series (from 1/2 to 1/4). Pretreatment of stimulated samples with SDS (0.02%) had no effect on the quantitative detection of IL-1*β*. For this reason, SDS is only used in unstimulated (whole saliva) samples, where it exerts its effect of dissociating mucin particles. The amounts of IL-1*β* and VEGF were determined in picograms/millimeter (pg/ml) by measurement of optical density (OD) on a plate reader using a wavelength of 450 nm. A standard curve generated from the OD values of standards provided by the manufacturer was used to determine the concentration of IL-1*β* and VEGF.

As the capacity to secrete saliva varies greatly between individuals, the total secretory output (pg/min) of IL-1*β* and VEGF was calculated by multiplying the flow rate (ml/min) for the respective individual by the concentration of IL-1*β* and VEGF (pg/ml) [19]. The mean output (pg/min) of IL-1*β* and VEGF was presented according to the different orthodontic treatment stages: alignment, space closure, and finishing.

### 2.5. Statistical Analysis

Arithmetic means and standard deviations were calculated for IL-1*β* and VEGF levels in the saliva for all treatment stages. Repeated measurement analysis of variance (ANOVA) was performed to compare the means of treatment stages (baseline, alignment, space closure, and finishing). The Tukey multiple comparison test was performed after the ANOVA, to determine the statistical significance between the treatment stages.

Bland-Altman plots were used for the interpretation of the percentage difference of IL-1*β* and VEGF between the baseline and alignment, baseline and space closure, and baseline and finishing, using 95% limits of agreement [20]. The statistical significance level was considered to be 5%, and statistics software (version 25; SPSS, Chicago, Ill) was used for all statistical computations.

## 3. Results

### 3.1. Demographic and Clinical Data

Table 1 shows the demographic data of the patients. The mean age of the patients at baseline was 18.4 ± 1.4. During the total treatment time (24.4 ± 5.4 months), between 24 and 38 saliva samples were analysed for each patient, depending on the treatment duration (Table 1). In total, 268 samples were analysed to determine the salivary output of IL-1*β* and VEGF.

### 3.2. Salivary Output of IL-1*β* in Stimulated Saliva

The levels of IL-1*β* in unstimulated saliva samples were close or below to the detection limit of ELISA (data not shown). Therefore, the IL-1*β* analyses were performed in stimulated saliva samples.

**Figure 1:**
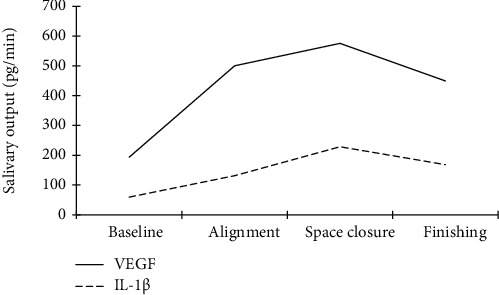
Mean salivary output (pg/min) of patients (*N* = 9) for IL-1*β* (dotted line) and VEGF (solid line) during the treatment stages: baseline, alignment, space closure, and finishing.

Figure 1 and Table 2 show a highly significant increase in the mean output of IL-1*β* at space closure (228.49 ± 112.54 pg/min), compared with the baseline (59.53 ± 48.23 pg/min), *p* = 0.002. Although a higher mean output was seen for the alignment (131.28 ± 70.97 pg/min) than for the baseline, the difference did not reach significance, *p* = 0.052. At finishing (168.10 ± 135.31 pg/min), the mean output of IL-1*β* decreased compared with space closure (*p* = 1.00; however, without reverting to the baseline values (*p* = 0.172).

### 3.3. Salivary Output of VEGF in Unstimulated Saliva

The levels of VEGF in stimulated saliva samples were close or below to the detection limit of ELISA (data not shown). Therefore, the VEGF analyses were performed in unstimulated saliva samples.

A significant increase in the mean output of VEGF was observed at space closure (575.27 ± 103.67 pg/min), compared with the baseline (193.33 ± 43.77 pg/min), *p* = 0.025 (Figure 1, Table 2). At alignment, a higher mean output was seen (550.21 ± 135.14 pg/min) than at the baseline, although without a significant difference, *p* = 0.414. The mean output of VEGF at finishing (449.23 ± 132.45 pg/min) decreased compared with space closure (*p* = 1.00); however, the values were still high and did not revert back to baseline values (*p* = 0.207).

### 3.4. Agreement between Treatment Stages for Salivary Output of IL-1*β* and VEGF

**Figure 2:**
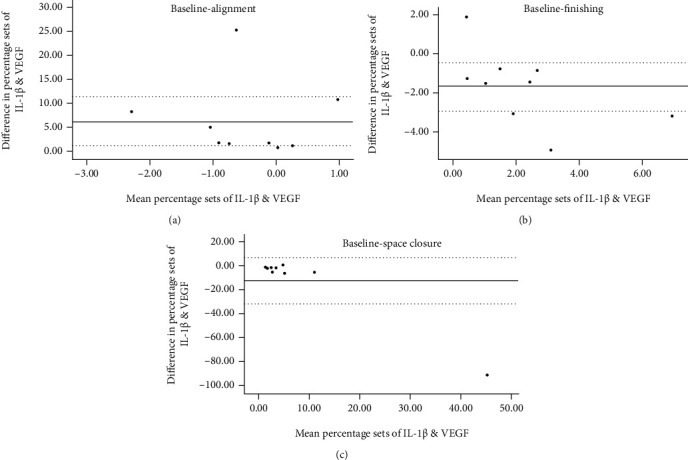
Bland-Altman plot comparing the percentage difference of IL-1*β* and VEGF between baseline and alignment (a), baseline and space closure (b), and baseline and finishing (c). The dotted line represents the upper and lower 95% limits of agreement, and the solid line represents the mean percentage difference between IL-1*β* and VEGF.

The mean percentage difference between the baseline and alignment IL-1*β* and VEGF was 6.24, with 95% limits of agreement of 1.06 to 11.41% (Figure 2(a)). Between the baseline and space closure, the mean percentage difference of IL-1*β* and VEGF was -12.66, with 95% limits of agreement of -31.93 to 6.61% (Figure 2(b)). For the baseline and finishing, the mean percentage difference of IL-1*β* and VEGF was -1.68 with 95% limits of agreement of -2.92 to -0.45% (Figure 2(c)).

## 4. Discussion

The present pilot study used the saliva as a research material to monitor the levels of VEGF and IL-1*β* change during 2 years of orthodontic tooth movement initiated by the orthodontic forces of a fixed appliance. To the far of our knowledge this is the first in vivo study in human settings using the saliva to monitor VEGF and IL-1*β* in tooth movement. The results of the study showed that a few weeks after the start of treatment, the levels of VEGF and IL-1*β* increased gradually and reached their highest levels at space closure, when horizontal forces were applied to yield bone remodelling. After the force was removed at the finishing stage, the levels of VEGF and IL-1*β* were almost halved; however, they were still higher than the baseline levels when analysed six to eight weeks after the space closure.

Continuous pressure to the periodontal ligament stimulates the production of VEGF and thereby angiogenesis [7]. In an experimental study on mice, VEGF has been shown to regulate bone metabolism and tooth movement through the activation of osteoclasts in the periodontal ligament [14]. During orthodontic treatment, compression forces initiate the formation of new blood vessels in the periodontium by activation of VEGF. This, in turn, stimulates vascularisation of new bone formation by angiogenesis, which is achieved by increased levels of VEGF [14]. Accordingly, the current study supports the idea that VEGF via osteoclast activation and angiogenesis perform functions in bone remodelling during orthodontic tooth movement, as the highest level of VEGF was found during space closure.

Physiological levels of VEGF are of great importance during bone repair in order to achieve a good result, where low levels have been shown to result in impaired healing [13]. However, very high levels of VEGF may also have harmful effects via inhibition of osteoblast maturation and bone mineralization. Moreover, sustained high levels of VEGF may activate osteoclast unnecessarily, which may result in resorption of the newly formed bone, whereas low plasma levels of VEGF in postmenopausal women have been associated with decreased oestrogen levels [21].

Another study in ovariectomised mice showed decreased levels of VEGF, further supporting the idea that hormonal factors may regulate the levels of VEGF as well as bone metabolism [22]. Furthermore, according to previous studies, there is a close relationship between osteoclast activity and oestrogen levels [23]. Low levels have been shown to accelerate tooth movement by increasing osteoclast activation and inducing bone resorption. In contrast, high levels have an inhibitory effect on tooth movement by increasing bone density and mineral content, which slows bone resorption [24]. In addition, the oestrogen level in plasma has been shown to correlate well with the level in the saliva [25]. In the present study, only young female patients who had their first menstrual period two years ago were included in order to exclude confounding factors such as gender and hormonal differences.

IL-1*β* is a proinflammatory cytokine and elevated levels are seen in different conditions as an inflammatory response [26]. Mechanical stress that occurs with force application leads to a sterile inflammation, which occurs during the process of bone remodelling in orthodontic treatment [11]. Bacteria-induced inflammation of the gingiva, so-called gingivitis, may also cause elevated levels of IL-1*β* in the saliva [27]. One of the inclusion criteria in this study was that patients should have optimal oral hygiene before starting treatment but also during the entire treatment period. All the patients in this study had good oral hygiene, except for three patients who showed mild gingivitis on three visits. Although the analysis of these samples showed no deviating values, they were still excluded from the study.

Bone remodelling during orthodontic tooth movement is suggested to be regulated, e.g., by cytokines that interact with bone cells directly or indirectly [15]. Orthodontic force application to the teeth leads to vascular changes in periodontal tissues, causing a sterile inflammation. As a result, proinflammatory molecules and leukocytes are released and migrate to the area of inflammation through the capillaries. IL-1*β*, which is a proinflammatory cytokine, is secreted by mononuclear cells in the early stages of induced mechanical stress [11]. Further, continued mechanical stress leads to increased levels of IL-1*β* in the areas of compression [28]. The IL-1*β* levels are closely correlated with osteoclast activation, which is crucial for the bone remodelling process and the speed of tooth movement [16]. This is in line with the results from the present study, where levels of IL-1*β* and VEGF started to increase already in the early phase of orthodontic treatment and then reached the maximum levels at space closure, where most of the continued compression forces are applied.

Another known signalling pathway for bone remodelling has been seen to be through RANK/RANKL interactions. In areas of mechanical stress, the levels of IL-1*β* and VEGF increase, which induces RANK/RANKL expression and activation of cells responsible for bone formation [13]. Studies show that in response to mechanical stress, osteoblasts increase bone resorption by upregulation of the VEGF and RANKL expressions [12]. In summary, both IL-1*β* and VEGF play important roles in bone remodelling through their effect on osteoblast and osteoclast activity [12, 13].

The lack of standardised laboratory protocols for analysis of saliva specimens [1] has made it difficult to perform quantitative measurements of small proteins such as EGF and IL-8 [19]. However, in the present study, we followed a methodology for detection of VEGF in unstimulated saliva, which we had previously shown would improve the accuracy and reproducibility of ELISA readings when sodium dodecyl sulphate (SDS), a detergent to dissolve mucin aggregates, was used [19]. However, pretreatment of stimulated saliva samples with SDS had no effect on the quantitative detection of IL-1*β* (data not shown). Therefore, SDS was only used in the pretreatment of unstimulated saliva to dissociate densely packed mucin aggregates, also known as micelles [29]. Low levels of VEGF were found in stimulated saliva, probably due to dilution of glandular proteins with water during high stimulation of paraffin chewing (data not shown). Therefore, the analysis of VEGF was performed in unstimulated saliva samples, where the main VEGF origin is from the salivary glands [6]. In contrast, analysis of IL-1*β* was performed in stimulated saliva samples, where the main supply of IL-1*β* present in the saliva is considered the gingival crevicular fluid [30] and can be gained in saliva by chewing [31].

Gingival crevicular fluid (GCF) could be used as a diagnostic and prognostic tool; however, there are several limitations, such as being too expensive and time consuming to be used in clinical settings [17]. On the other hand, saliva testing is simple, inexpensive, and easy to apply in clinics and enables monitoring of cytokines and growth factors originated from GCF and salivary glands. The present study showed that IL-1*β* and VEGF can be monitored during orthodontic treatment and likely reflect the biological activity that occurs during orthodontic tooth movement and bone remodelling. The results from our study also show that the saliva as a biological fluid may be used to diagnose and monitor biological responses, as presented during bone remodelling in orthodontic treatment.

Since this is a prospective pilot study it has limitations, such as the small number of included subjects. The study was designed to collect saliva continuously during two years of orthodontic treament, which restricted the size of the study population. However, the results of this novel approach provide indications on how and when to sample saliva during orthodontic treatment. This is beneficial for future studies that may include larger study populations but with fewer saliva samples. It is important to get a better understanding of the biological processes that occur during orthodontic tooth movement.

## 5. Conclusions

This prospective pilot study is the first *in vivo* study in human settings using continuous sampling of the saliva to monitor VEGF and IL-1*β* during two years of orthodontic treatment. The salivary levels of both IL-1*β* and VEGF increased following orthodontic treatment and reached their peaks during space closure. This new knowledge shows how the levels of VEGF and IL-1*β* change during the different stages of an treatment, indicating their role in bone remodelling.

## Figures and Tables

**Table 1:** Treatment duration of the different treament stages and spacings in the upper and lower jaw (UJ, LJ) before start of alignment and space closure.

Baseline		Alignment			Space closure			Finishing	Total treatment time (months)
Crowding (mm), Mean ± SD	Overjet (mm), Mean ± SD	Spaces (mm), Mean ± SD		Treatment time (months), Mean ± SD	Spaces (mm), Mean ± SD		Treatment time (months), Mean ± SD	Treatment time (months), Mean ± SD	Mean ± SD
		UJ	LJ		UJ	LJ			
3.9 ± 2.4	6 ± 1.7	14.7 ± 1.2	14.7 ± 1.2	7.3 ± 1.7	7.3 ± 2.1	8.8 ± 2.9	13 ± 4.3	4.1 ± 2.1	24.4 ± 5.4

**Table 2:** Mean salivary output (pg/min) of patients (*N* = 9), 95% confidence interval, and significant values for IL-1*β* and VEGF during the treatment stages: baseline, alignment, space closure, and finishing. Comparisons by multivariate analysis of ANOVA: ^∗^*p* < 0.05, ^∗∗^*p* < 0.01.

	IL-1*β*				VEGF			
	Mean ± SD	95% confidence interval for mean		Analysis of variance	Mean ± SD	95% confidence interval for mean		Analysis of variance
		Lower bound	Upper bound			Lower bound	Upper bound	
Baseline (B)	59.5 ± 48.2	22.4	96.6	0.002^∗∗^ B/SC	193.3 ± 131.3	92.4	294.3	0.025^∗^ B/SC
Alignment (A)	131.3 ± 70.9	76.7	185.8	NS	500.2 ± 405.4	188.6	811.8	NS
Space closure (SC)	228.5 ± 112.5	141.9	315.0	0.002^∗∗^ SC/B	575.3 ± 311.0	336.2	814.3	0.025^∗^ SC/B
Finishing (F)	168.1 ± 135.3	64.0	272.1	NS	449.2 ± 397.4	143.8	754.7	NS

## Data Availability

The data used to support the findings of this study are included within the article.
